# Quantitative 3D correlative light and electron microscopy of organelle association during autophagy

**DOI:** 10.1247/csf.22071

**Published:** 2022-11-22

**Authors:** Satoru Takahashi, Chieko Saito, Ikuko Koyama-Honda, Noboru Mizushima

**Affiliations:** 1 Department of Biochemistry and Molecular Biology, Graduate School of Medicine, The University of Tokyo, Tokyo, Japan; 2 Department of Neurosurgery, Graduate School of Medical and Dental Sciences, Tokyo Medical and Dental University, Tokyo, Japan

**Keywords:** 3D-CLEM, autophagosome, electron microscopy, endoplasmic reticulum, lysosome

## Abstract

In macroautophagy, disk-shaped double-membrane structures called phagophores elongate to form cup-shaped structures, becoming autophagosomes upon closure. These autophagosomes then fuse with lysosomes to become autolysosomes and degrade engulfed material. Autophagosome formation is reported to involve other organelles, including the endoplasmic reticulum (ER) and mitochondria. Organelles are also taken up by autophagosomes as autophagy cargos. However, few studies have performed systematic spatiotemporal analysis of inter-organelle relationships during macroautophagy. Here, we investigated the organelles in contact with phagophores, autophagosomes, and autolysosomes by using three-dimensional correlative light and electron microscopy with array tomography in cells starved 30 min. As previously reported, all phagophores associate with the ER. The surface area of phagophores in contact with the ER decreases gradually as they mature into autophagosomes and autolysosomes. However, the ER still associates with 92% of autophagosomes and 79% of autolysosomes, suggesting that most autophagosomes remain on the ER after closure and even when they fuse with lysosomes. In addition, we found that phagophores form frequently near other autophagic structures, suggesting the presence of potential hot spots for autophagosome formation. We also analyzed the contents of phagophores and autophagosomes and found that the ER is the most frequently engulfed organelle (detected in 65% of total phagophores and autophagosomes). These quantitative three-dimensional ultrastructural data provide insights into autophagosome–organelle relationships during macroautophagy.

## Introduction

**Fig. 1:**
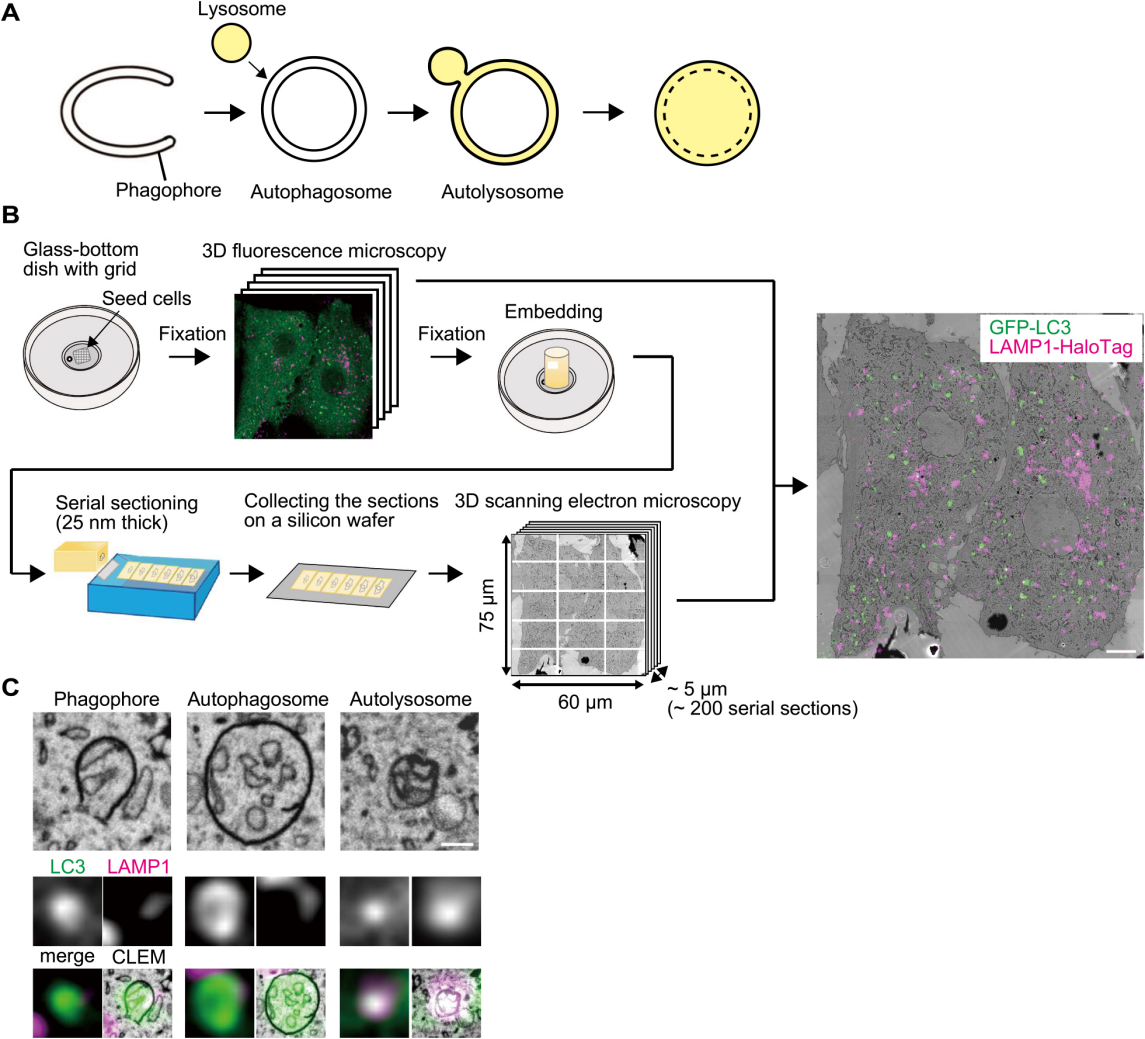
3D-CLEM procedure with array tomography (A) A model of autophagosome and autolysosome formation. (B) Schematic representation of the procedure for 3D-correlative light and electron microscopy (CLEM) with array tomography. A representative CLEM image of mouse embryonic fibroblasts (MEFs) stably expressing GFP-LC3 (green) and LAMP1-HaloTag (magenta) is shown. Scale bar, 5 μm. (C) Examples of a phagophore, autophagosome, and autolysosome are shown. Scale bar, 0.2 μm.

In macroautophagy, disk-shaped double-membrane structures called phagophores (also known as isolation membranes) elongate and bend to form cup-shaped structures (Fig. 1A). Upon closure of the rim, the phagophores become spherical structures known as autophagosomes. These autophagosomes fuse with lysosomes to become autolysosomes and then degrade the sequestered cytoplasmic material with lysosomal enzymes (Mizushima and Komatsu, 2011; Nakatogawa, 2020; Søreng *et al.*, 2018).

Because autophagosomes are formed de novo, their formation relies on the import of lipids and other components from other organelles. Previous reports showed that autophagosome formation undergoes in contact with the endoplasmic reticulum (ER) (Axe *et al.*, 2008; Biazik *et al.*, 2015; Bieber *et al.*, 2022; Gudmundsson *et al.*, 2022; Hayashi-Nishino *et al.*, 2009; Uemura *et al.*, 2014; Ye *et al.*, 2022; Ylä-Anttila *et al.*, 2009), ER exit sites (Biazik *et al.*, 2015), mitochondria (Biazik *et al.*, 2015; Bieber *et al.*, 2022; Hailey *et al.*, 2010), ER-mitochondria contact sites (Hamasaki *et al.*, 2013), the Golgi apparatus (Biazik *et al.*, 2015; Nishida *et al.*, 2009), lipid droplets (Bieber *et al.*, 2022; Dupont *et al.*, 2014; Ogasawara *et al.*, 2020), endosomes (Biazik *et al.*, 2015), and the nuclear envelope (Bieber *et al.*, 2022; English *et al.*, 2009). These organelles form contacts with phagophores and can communicate with them through vesicular or non-vesicular transfer. For example, ATG2 transfers lipids from the ER to phagophores (Maeda *et al.*, 2019; Osawa *et al.*, 2019; Valverde *et al.*, 2019). Other organelle contacts may also provide (or exchange) some components to phagophores or autophagosomes. These organelle contacts are not static. Although autophagosomes are not highly mobile during the formation process, they become so after formation (Kimura *et al.*, 2008; Maday and Holzbaur, 2014; Pankiv *et al.*, 2010). It has been suggested that autophagosomes dissociate from the ER after closure (Hayashi-Nishino *et al.*, 2009). It is possible that autophagosomes stop incorporating lipids from the ER and move toward lysosomes. However, when and how the ER detaches from autophagosomes has not been systematically investigated. In addition, organelles can be sequestered inside autophagosomes as cargos. These functions are collectively called organellophagy and includes mitophagy and ER-phagy (Gatica *et al.*, 2018; Mizushima, 2022; Okamoto, 2014). However, the relative frequency of the engulfment of each organelle during starvation is not fully understood.

Studies that systematically investigated the inter-organelle relationships during autophagy have been limited. One of the reasons is that the resolution of fluorescence microscopy is not adequate to accurately assess organelle contacts and sequestration. Conventional two-dimensional electron microscopy (EM) can detect organelle contacts but small-scale contacts are easily overlooked. Furthermore, it can be difficult to determine whether an autophagosome is indeed closed. Three-dimensional EM has been used to overcome these limitations. Electron tomography previously revealed tight and extensive contacts of phagophores with the ER (Biazik *et al.*, 2015; Hayashi-Nishino *et al.*, 2009; Uemura *et al.*, 2014; Ye *et al.*, 2022; Ylä-Anttila *et al.*, 2009). Contacts with putative ER exit sites and Golgi cisternae and connections to endolysosome-related structures have also been observed (Biazik *et al.*, 2015). Another recent study using cryo-electron tomography showed fine structures of the contacts of phagophores and autophagosomes with other organelles such as the vacuole, the ER, the nucleus, lipid droplets, and mitochondria in yeast cells (Bieber *et al.*, 2022). However, electron tomography can capture images with a thickness of only several hundred nanometers, which is thinner than that of typical autophagosomes. Therefore, it remains a challenge to distinguish between closed and unclosed structures with this method. Serial block-face scanning electron microscopy (SBF-SEM) and array tomography are ideal methods for collecting entire autophagy-related structures from large areas, covering entire cells from bottom to top (Denk and Horstmann, 2004; Micheva and Smith, 2007). In SBF-SEM, an ultramicrotome is installed in a scanning electron microscope and serial section images (40-nm slices) are collected. Biazik *et al.* used SBF-SEM and found that phagophores contact the ER (100%), mitochondria (29%), endosomes or lysosomes (17%), the Golgi complex (0.5%), and ER exit sites (0.5%) in starved NRK-52E cells (Biazik *et al.*, 2015). More recently, Gudmundsson *et al.* used live-cell imaging and SBF-SEM and showed that phagophore precursors emerged close to the ER (Gudmundsson *et al.* 2022). However, how these contacts change after closure and lysosome fusion and whether these organelles are also engulfed were not investigated.

In the present study, we conducted three-dimensional correlative light and electron microscopy (3D-CLEM) with array tomography, in which serial sections were manually prepared and observed by SEM (Koike and Yamada, 2019; Micheva and Smith, 2007). We systematically analyzed the organelle contacts of phagophores, autophagosomes, and autolysosomes and described how they change during maturation in mouse embryonic fibroblasts (MEFs) starved 30 min. We also quantitatively determined the organelles that are engulfed by autophagosomes.

## Materials and Methods

### Cell line and culture conditions

MEFs were cultured in Dulbecco’s modified Eagle medium (DMEM) (D6546, Sigma-Aldrich) supplemented with 10% fetal bovine serum (FBS) (173012, Sigma-Aldrich) and 2 mM l-glutamine (25030-081, GIBCO) in a 5% CO_2_ incubator at 37°C.

### Plasmids

Plasmids for stable expression in MEFs were generated as follows: DNA fragments encoding enhanced GFP, rat LC3B (Kabeya *et al.*, 2000), HaloTag7 (HaloTag) (N2701, Promega), and rat LAMP1 (Tsuboyama *et al.*, 2016) were inserted into the retroviral plasmid pMRX-IB (Morita *et al.*, 2018) and pMRX-IP (Saitoh *et al.*, 2002) by using the seamless ligation cloning extract (SLiCE) method.

### Stable expression in MEFs by retrovirus infection

To prepare the retrovirus solution, HEK293T cells were transfected for 4 to 6 h with the pMRX-IB-based or pMRX-IP-based retroviral plasmid, pCG-gag-pol, and pCG-VSV-G (a gift from Dr. T. Yasui, National Institutes of Biomedical Innovation, Health and Nutrition) by using Lipofectamine 2000 (11668019, Thermo Fisher Scientific) followed by replacement of the medium with DMEM. After 2 to 3 days, the retrovirus-containing medium was harvested, filtered with a 0.45-μm filter unit (Ultrafree-MC, Millipore), and added to MEFs with 8 μg/mL polybrene (H9268, Sigma-Aldrich). The following day, selection was performed with 2–3 μg/mL blasticidin (022-18713, Fujifilm Wako Pure Chemical Corporation) or 1–2 μg/mL puromycin (P8833, Sigma-Aldrich).

### 3D-CLEM

A glass base dish with 150-μm grids (TCI-3922-035R-1CS, Iwaki) was pretreated as follows. A glass cover slip was coated with carbon by a vacuum evaporator (IB-29510VET, JEOL), and treated with 0.1% gelatin for 15 min under ultraviolet irradiation. Then, MEF cells were cultured in the pretreated glass base dish for 2 days. Cells were incubated with 200 nM SF650-conjugated Halo ligand (A308-02, GoryoChemical) in DMEM supplemented with 10% FBS and 2 mM l-glutamine 20 min before imaging. The cells were washed with amino acid-free and FBS-free DMEM (048-33575, Wako Pure Chemical Industries) and incubated for 30 min in the medium to induce autophagy by starvation. After starvation, the cells were washed twice with 0.1 M phosphate buffer pH 7.4 (RM102-5L, LSI Medience) and fixed with 2% paraformaldehyde (26126-54, Nacalai Tesque) and 0.5% glutaraldehyde (G018/1, TAAB) in 0.09 M phosphate butter pH 7.4 for 1 h at 4°C. The cells were washed three times with 0.1 M phosphate buffer pH 7.4 and Z-section images (350-nm each) were acquired via the confocal microscope system (FV3000, Olympus) equipped with a 60^x^ oil-immersion objective lens (NA 1.4, PLAPON60XOSC2, Olympus).

After fluorescence microscopy, the cells were fixed with 2.5% glutaraldehyde (G018/1, TAAB) in 0.1 M cacodylate buffer pH 7.4 (37237-35, Nacalai Tesque) overnight and fixed with 1% osmium tetroxide (3020-4, Nisshin EM) and 1.5% potassium ferrocyanide (161-03742, Wako) in 0.065 M cacodylate buffer for 2 h at 4°C, washed five times with Milli-Q water, treated with 3% uranium acetate for 1 h, and dehydrated in an ascending series of ethanol. After 1-h dehydration in 100% ethanol, the cells were embedded in Epon (EPON 812, TAAB), polymerized at 40°C for 12 h, and polymerized at 60°C for 48 h. The blocks were trimmed to ~100 × 100 μm cuboids using razor blades. To create ultra-thin serial sections, we used a diamond knife with an ultra-jumbo boat (Ultrajumbo 35 degree, Diatome) mounted on an ultramicrotome (UC7, Leica). Sections were cut at 25-nm thickness to create a ribbon of 100–200 serial sections and transferred to a silicon wafer (Mitsubishi Materials Trading Corporation), which is held and manipulated by a micromanipulator (Märzhäuser Wetzlar). Samples were stained with 3% uranium acetate and Reynolds’ lead citrate.

Scanning electron microscopic (SEM) images were acquired using an electron microscope (JSM7900F, JEOL) following three steps. First, based on the photograph of a wafer with serial sections, SEM images with magnification ×100 for all serial sections (each image size is 750 μm × 1000 μm) were acquired. Second, based on the SEM images from the first step, SEM images with a magnification of ×300 were acquired for more precise information about all serial sections (each image size is 250 μm × 333 μm). Finally, based on the SEM images from the second step, SEM images with magnification ×5000 for targeted cells (each image size is 15 μm × 20 μm) were acquired. All steps were performed semi-automatically using Array Tomography Supporter 1.0.0.0 software (System In Frontier). SEM images were automatically tiled in order by Measurement Advisor 4.1.14.0 software (System In Frontier). Images were stacked in order using Stacker NEO TEMography.com 3.3.4.0 software (System In Frontier Inc.) and Fiji software (ImageJ 2.9.0; National Institute of Health) (Schindelin *et al.*, 2012). Three-dimensional reconstruction of EM images was created using Image-pro 10.0.12 (Media Cybernetics).

The ER and mitochondria were defined based on the morphology in 3D observation. The ER was identified as tubular structures with a single membrane having less electron density compared with autophagosomal membranes, and with continuity to ribosome-bound membranes. Mitochondria were identified as organelles with cristae.

### Measurement of the intermembrane distance between autophagic structures and organelles

From the EM images, the line profile including both the outer autophagosomal membrane and organelle membrane of interest was created using Fiji software. The minimum distance was determined by measuring the length between the points with local minimum-gray values in organelle membranes.

## Results

### Characterization of LC3-positive structures by 3D-CLEM using array tomography

To classify phagophores, autophagosomes, and autolysosomes in 3D-CLEM, we used MEFs stably expressing GFP-LC3B (hereafter referred to simply as “GFP-LC3”) as an autophagosome marker and LAMP1-HaloTag as a lysosome marker. After starvation for 30 min, cells were fixed with paraformaldehyde and glutaraldehyde, and imaged by fluorescence microscopy. Then, the cells were fixed using glutaraldehyde and further fixed with reduced osmium for EM. Epoxy resin-embedded samples were cut at 25-nm thickness, and the resultant serial sections were observed by array tomography (Fig. 1B). We identified a total of 131 complete 3D structures of GFP-LC3-positive autophagic structures from 3D-EM images of two nearly-whole cells. These structures included 28 phagophores (open structures), 79 autophagosomes (LAMP1-HaloTag-negative closed structures), and 24 autolysosomes (LAMP1-HaloTag-positive closed structures) (Fig. 1C).

**Fig. 2:**
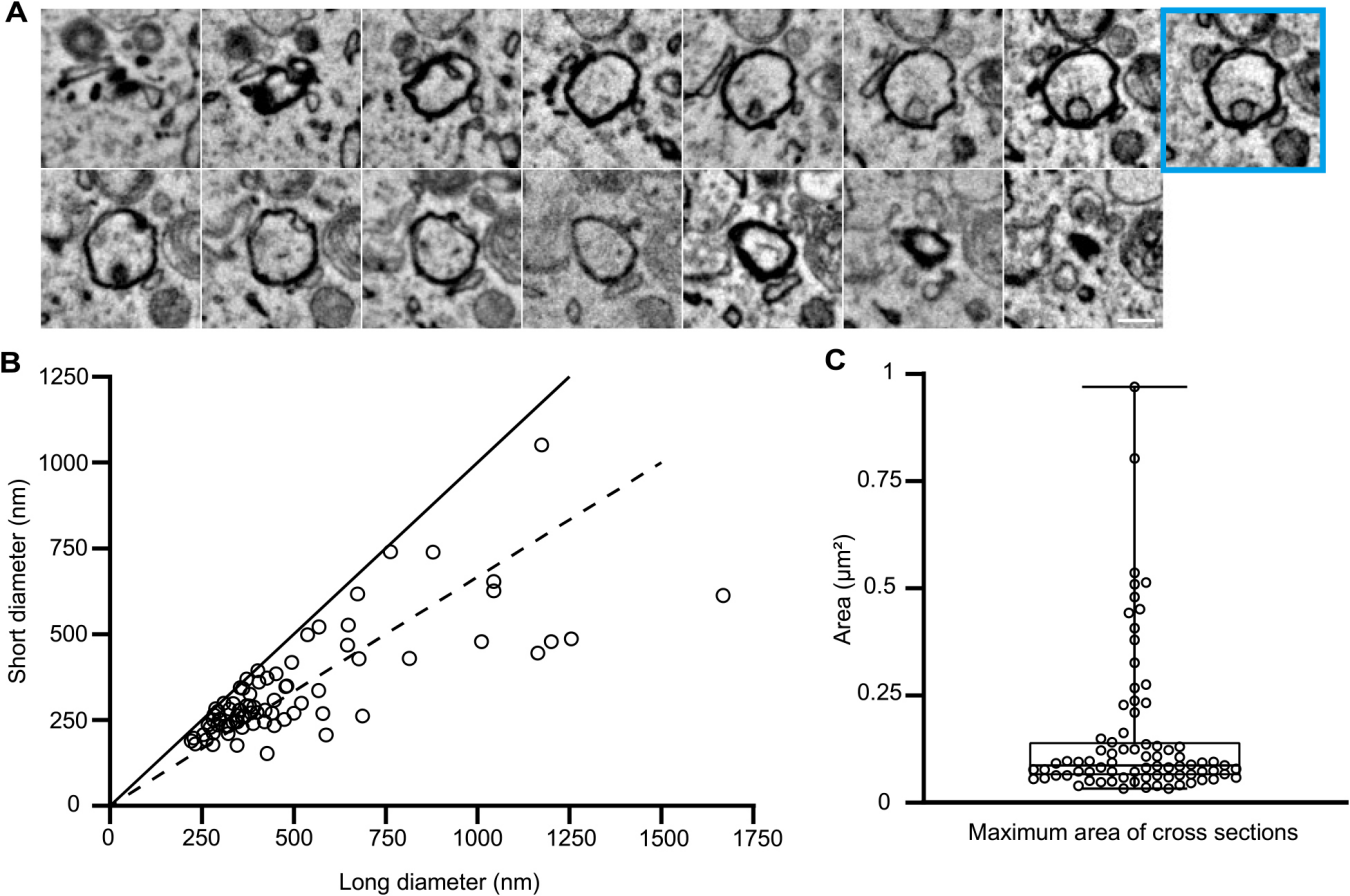
Distribution of autophagosomal diameters and maximum areas (A) Serial EM images (25-nm thickness) of an autophagosome are shown. The section with the maximum area is indicated by a cyan square. Scale bar, 0.2 μm. (B) Distribution of the short and long diameters in the maximum areas of 79 autophagosomes. The closer the plots are to the line, the more spherical the autophagosomes appear. The dotted line indicates where the long diameters are 1.5× greater than the short diameters. (C) The maximum area of cross-sections was calculated based on the long and short diameters. In the box plot, a solid bar indicates median, a box indicates the interquartile range (25th to 75th percentile), and whiskers indicate the largest and smallest values.

Using the 3D-CLEM images, we first analyzed the sizes and shapes of autophagosomes (GFP-LC3 positive and LAMP1-HaloTag negative). To this end, the maximum long and short diameters were measured (Fig. 2A). As previously reported, the outer and inner autophagosomal membranes tightly attach to each other, and this is maintained during the sample preparation for EM when reduced osmium is used (Uemura *et al.*, 2014). The long diameter ranged from 221 to 1668 nm with a median of 389 nm, and the short diameter ranged from 189 to 613 nm with a median of 279 nm (Fig. 2B). Most autophagosomes were spherical, but about 30% (25/79) were ellipsoid with a long diameter 1.5× greater than the short diameter. The maximum area ranged from 0.033 to 0.97 μm^2^ with a median of 0.088 μm^2^ (Fig. 2C).

### Quantitative analysis of organelle contacts during autophagy

**Fig. 3:**
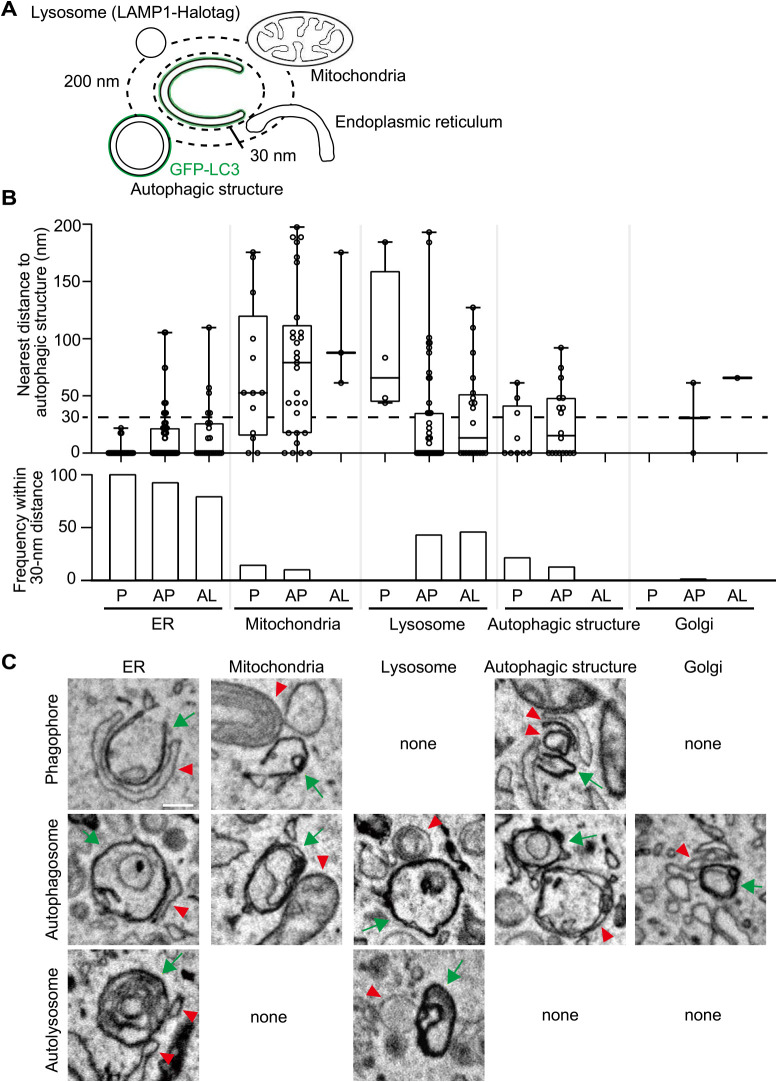
Relationship between autophagic structures and other organelles (A) The nearest distance between autophagic structures and other organelles was measured in 3D-EM images. The autophagic structures (GFP-LC3 positive) and lysosomes (LAMP1-Halotag positive) were identified in 3D-CLEM images. Other organelles were identified based on their morphology. (B) From two MEFs, 28 phagophores, 79 autophagosomes, and 24 autolysosomes were collected. The upper graph indicates the nearest distance between autophagic structures and other organelles. The distance was measured when the organelle localized within 200 nm. The dotted line indicates a 30-nm distance. In the box plots, solid bars indicate medians, boxes indicate the interquartile range (25th to 75th percentile), and whiskers indicate the largest and smallest values. The lower graph indicates the frequency for each organelle observed within a 30-nm distance from autophagic structures. P, phagophore; AP, autophagosome; and AL, autolysosome. (C) Representative EM images of autophagic structures in contact with other organelles. Green arrows indicate phagophores, autophagosomes, or autolysosomes. Red arrowheads indicate the organelles in contact with the autophagic structures. Scale bar, 0.2 μm.

To investigate organelle contacts during autophagy, we first identified organelles within 200 nm of the autophagic structures and measured the minimum distances (Fig. 3A). Organelle contact sites are often defined as sites where two membranes are within 10–30 nm of each other (Achleitner *et al.*, 1999; Biazik *et al.*, 2015; Scorrano *et al.*, 2019). We measured the ratio of various organelles found within 30 nm of autophagic structures. The ER was observed for all phagophores (28/28), most (92%) autophagosomes (73/79), and even 79% of autolysosomes (19/24) (Fig. 3B, C). Out of the 28 phagophores, 24 were attached to the ER at the rim of phagophores, while 27 were on the outer membrane (side and back). Mitochondria were observed for 14% (4/28) of the phagophores, 10% (8/79) of the autophagosomes, and none of the autolysosomes (Fig. 3B, C). The Golgi apparatus was rarely observed next to autophagic structures. Lipid droplets, the nuclear membrane, and the plasma membrane were not observed within the 30-nm range of any autophagic structure. These results suggest that autophagosomes are always generated on the ER and remain there for a while, maintaining contact with the ER. Lysosomes were not observed within the 30-nm range from phagophores (0/28) but were observed from 43% (34/79) of autophagosomes and 46% (11/24) of autolysosomes (Fig. 3B, C). Contacts with other organelles were only occasionally observed, implying that they may not be essential for autophagosome formation during early starvation. As we observed chemically fixed cells (2% paraformaldehyde and 0.5% glutaraldehyde as fixation for fluorescence microscopy, 2.5% glutaraldehyde as primary fixative, and potassium ferrocyanide and osmium as secondary fixative) by SEM, the IMAT (isolation membrane associated tubule) might not be preserved as previously described (Uemura *et al.*, 2014).

Autophagic structures were sometimes observed close to each other. Autophagic structures were observed within the 200-nm range of 41% (9/24) and within the 30-nm range of 25% (6/24) of phagophores. This ratio decreased to 23% (18/79, 200-nm range) and 13% (10/79, 30-nm range) for autophagosomes and zero for autolysosomes (Fig. 3B, C). These results suggest that there are potential hot spots for autophagosome formation, and once formed, autophagosomes disperse in the cytosol.

### Gradual dissociation of autophagosomes and autolysosomes from the ER

**Fig. 4:**
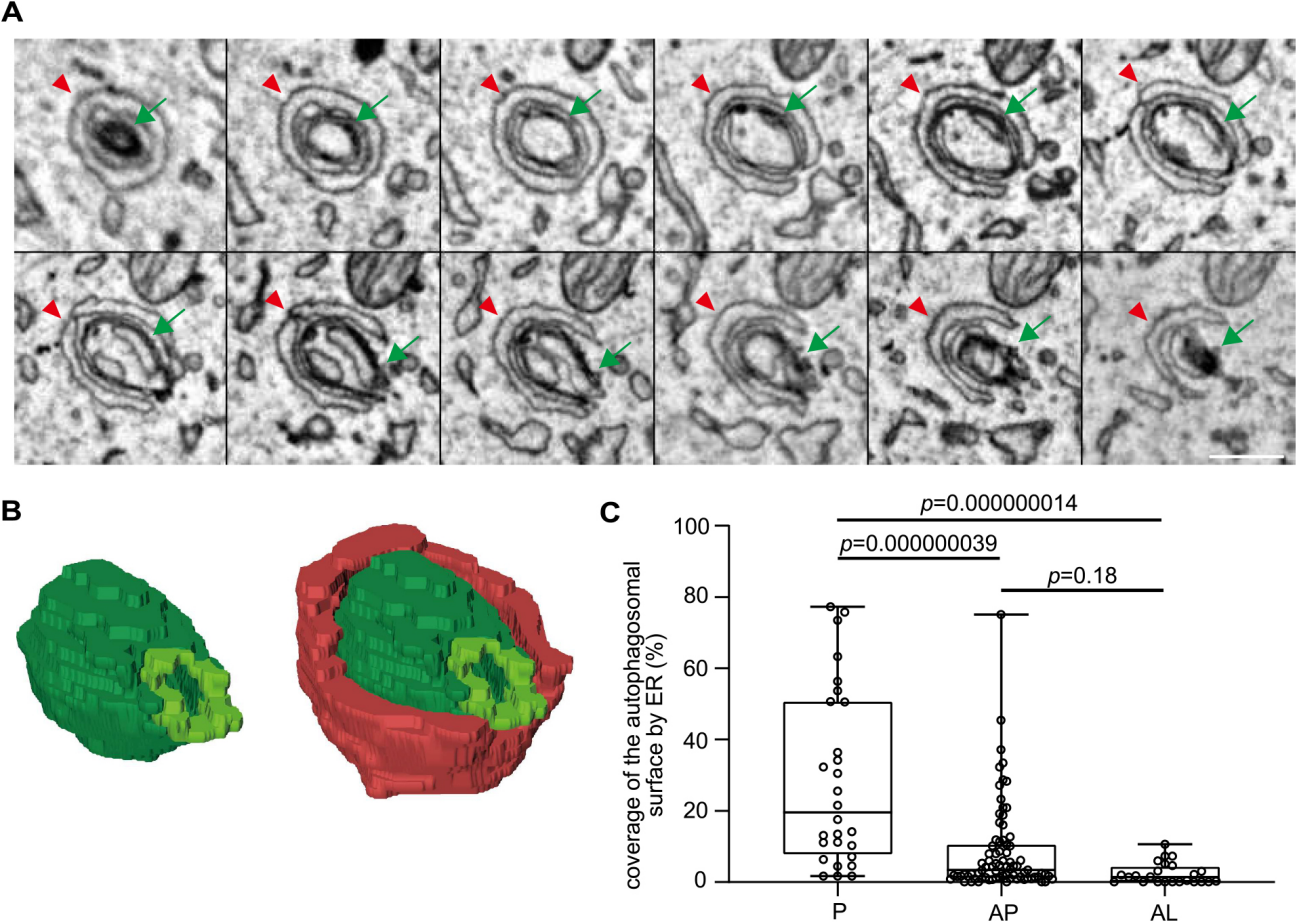
Gradual detachment of the ER from autophagosomes and autolysosomes (A) Serial EM images showing the ER contacting the rim and the outer and inner membranes of a phagophore. Green arrows indicate a phagophore and red arrowheads indicate the ER. Scale bar, 0.2 μm. (B) 3D reconstruction of the images in (A). Green indicates the phagophore, yellow green indicates the rim of the phagophore, and red indicates the ER in contact with the phagophore (74% of the autophagosomal surface is covered by ER in this example). (C) Percentages of the autophagosomal outer surface area in contact with the ER. The percentages were calculated based on serial EM images of whole autophagic structures. The contact sites were defined as the areas where the ER and autophagic membranes were present within 30 nm. P, phagophore; AP, autophagosome; and AL, autolysosome. Solid bars indicate medians, boxes indicate the interquartile range (25th to 75th percentile), and whiskers indicate the largest and smallest values. Differences were statistically analyzed by one-way ANOVA and Tukey’s multiple comparison test.

We next quantified how much of the autophagosomal surface was surrounded by the ER. We measured the total lengths of the outer surface and the length of the portion in contact with the ER in each serial EM image and calculated the percentage of the ER-contacting autophagosomal surface (Fig. 4A, B). The ER covered 28.6% (average) and 19.5% (median) of the outer surface of phagophores, 8.6% (average) and 3.5% (median) of autophagosomes, and 2.4% (average) and 1.5% (median) of autolysosomes (Fig. 4C). The area surrounded by the ER was greater in phagophores than in autophagosomes and autolysosomes. These data suggest that contact with the ER gradually decreases during autophagosome maturation and only minor contacts remain after becoming autolysosomes.

### Organelles in phagophores and autophagosomes

**Fig. 5:**
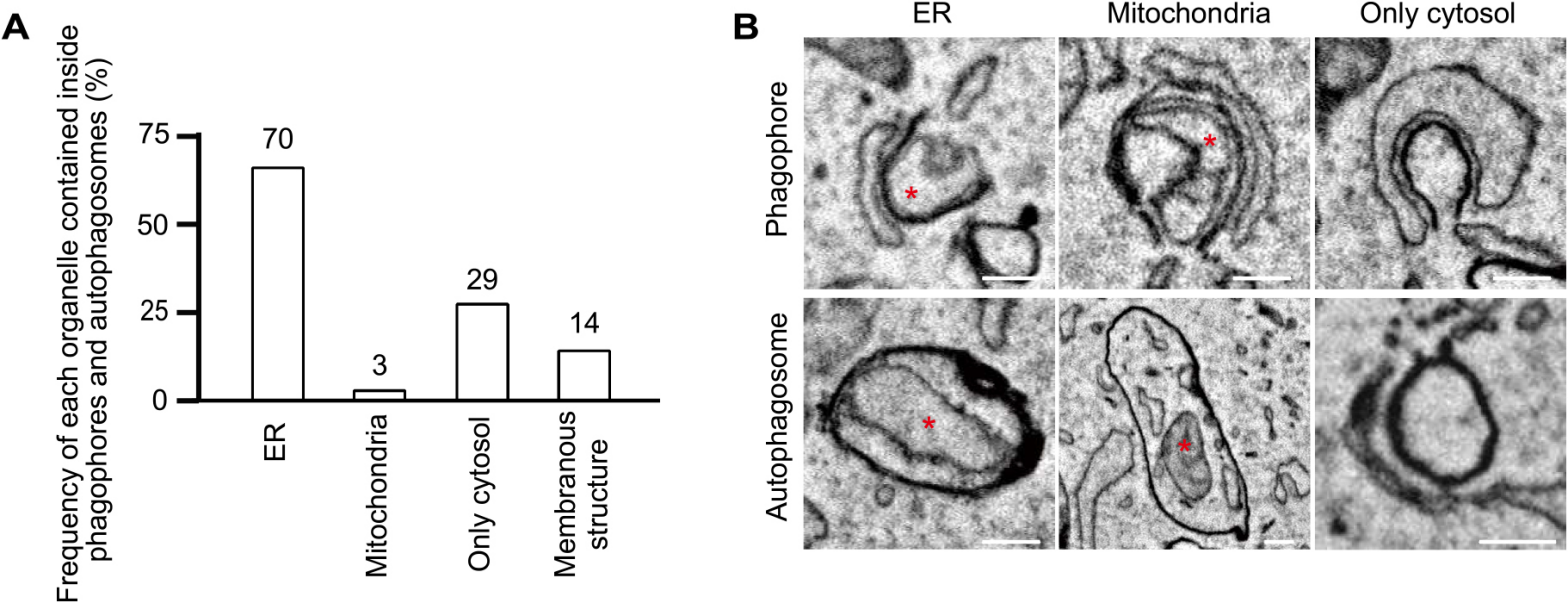
Autophagosomal contents in phagophores and autophagosomes (A) From 3D-EM images of 28 phagophores and 79 autophagosomes (total 107), the organelles inside were defined based on morphology. ER fragments were identified as ribosome-bound organelles, tubular structures with a single membrane having less electron density compared with autophagosomal membranes, or organelles connected to the ER outside. Mitochondria were identified as organelles with cristae. “Membranous structures” indicate organelles not specified as either the ER or mitochondria. (B) The representative EM images of phagophores and autophagosomes containing indicated organelles (red asterisks). Scale bar, 0.2 μm.

We next investigated the organelles observed within the autophagic structures. We examined only phagophores and autophagosomes because organelles undergoing degradation inside autolysosomes are difficult to identify. The engulfed organelles were identified based on their morphology. Of the 28 phagophores and 79 autophagosomes (total 107), 65% (70/107) contained ER fragments, 3% (3/107) contained mitochondria, and 27% (29/107) contained no membranous structures (only cytosol) (Fig. 5A, B). In addition, 13% (14/107) contained some undefined membranous structures, some of which were autophagosome-like structures (judging from the high electron density of their membranes). Thus, the most frequently engulfed organelle during 30-min starvation was the ER. The fact that about one-fourth of the phagophores and autophagosomes contained only the cytosol suggests that non-selective autophagy is dominant at an early phase of starvation.

## Discussion

In this study, we systematically quantified autophagosome–organelle contacts and autophagosomal contents by using 3D-CLEM with array tomography. This is a non-biased comprehensive study given that we analyzed all autophagic structures labeled with GFP-LC3 in nearly whole cells. In this study, we chose a starvation time of 30 min because we sought to capture images of structures at all stages, including phagophores.

The size of autophagosomes in MEFs starved 30 min was highly variable; their long diameter ranged from approximately 200 nm to 1700 nm with a median of 389 nm. These values may be smaller than those usually observed by EM (Eskelinen, 2005; Kovács *et al.*, 2007; Yamamoto *et al.*, 2020). This might be because we quantified all LC3-positive autophagosomes using 3D-CLEM in nearly whole cells, enabling us to detect small autophagosomes that would be difficult to find using only conventional EM (without fluorescence information). In addition, the autophagosomal size may be relatively small during early starvation. The size variation in MEFs starved 30 min appears to be larger than that reported in *Saccharomyces cerevisiae* (400–900 nm) (Takeshige *et al.*, 1992). The size of autophagosomes may differ depending on the starvation period, autophagy-inducing conditions, and cell types.

We confirmed that the ER associates with both the rim and the outer membrane of phagophores, as previously reported in studies using mammalian cells (Biazik *et al.*, 2015; Gudmundsson *et al.*, 2022; Hayashi-Nishino *et al.*, 2009; Kovács *et al.*, 2007; Uemura *et al.*, 2014; Ylä-Anttila *et al.*, 2009). This differs from the case in yeast, where the ER contacts mostly the rim (Bieber *et al.*, 2022). The ER at the rim may mediate lipid transfer because ATG2 is present there both in yeast and mammalian cells (Sakai *et al.*, 2020; Suzuki *et al.*, 2013). The role of the ER attaching to the outer membrane of phagophores and autophagosomes is not presently known. This attachment is observed not only in mammalian cells but also in *Caenorhabditis elegans* (Zhang *et al.*, 2015) and *Drosophila* (Strunov *et al.*, 2022). Thus, it should have an important function, but its importance might have been reduced during evolution in yeast. Given that autophagosomes are generated near the vacuolar membrane in yeast (Bieber *et al.*, 2022), the vacuolar membrane may have a similar function.

ER attachment to autophagosomes is reversible, and it has been reported that most autophagosomes remove ER membranes from their surface (Hayashi-Nishino *et al.*, 2009). However, our 3D study revealed that 92% of even closed autophagosomes and 8% of the autophagosomal surface still associated with the ER. These results suggest that autophagosomes maintain contact with the ER for a while rather than immediately dissociating. ER attachment was still detected on 79% of autolysosomes (2% of the autolysosomal surface), indicating that most autophagosomes are in the process of dissociation from the ER when they fuse with lysosomes. Although it has been suggested that autophagosomes and lysosomes move on microtubules and the directional movement of both organelles is important for efficient fusion between them (Zhao *et al.*, 2021), dissociation from the ER may not be necessary for the fusion with lysosomes.

Mitochondria were associated with 14% of phagophores, but only on the outer membrane, not at the rim. These associations were previously observed by SBF-SEM, but somewhat more frequently (29%) in NRK cells starved 1 h (Biazik *et al.*, 2015). The ER and mitochondria often attach to each other, forming the mitochondria-associated ER membrane (MAM). The MAM was previously reported to be involved in autophagosome formation (Hamasaki *et al.*, 2013). In our study, although the MAM was detected close to the phagophore, we could not detect a direct association between phagophores and the MAM. Lipid droplets are also reported to associate with autophagosomes (Bieber *et al.*, 2022; Dupont *et al.*, 2014; Ogasawara *et al.*, 2020). However, lipid droplets were too rare in the conditions we used in this study to enable quantitative analysis.

In this study, we found that autophagosomes are often generated near other autophagic structures and we hypothesized that there are potential hot spots for the formation of autophagosomes. In yeast, autophagosomes are always generated at the pre-autophagosomal structure (PAS, also called phagophore assembly site) near the vacuolar membrane, and therefore the PAS is definitely a hot spot for autophagosome formation. The PAS is a liquid-like condensate formed by liquid–liquid phase separation and includes various Atg proteins to initiate autophagosome formation (Fujioka *et al.*, 2020). If similar structures are present in mammalian cells, autophagosomes may be formed sequentially at the specific sites.

We found that the ER is most frequently sequestered among autophagy cargos. Because autophagosomes are always generated on the ER, it may be difficult for ER fragments to escape from engulfment by autophagosomes. In fact, the ER membranes in contact with the outer autophagosomal membranes are often connected to ER membranes inside autophagosomes through the rim (Hayashi-Nishino *et al.*, 2009). Thus, it is uncertain whether ER-phagy during acute starvation can be considered as selective autophagy or if it has any specific physiological meaning. Mitophagy is observed much less frequently, which is consistent with previous reports showing that mitochondria elongate during starvation and escape from autophagic degradation (Gomes *et al.*, 2011; Rambold *et al.*, 2011). Prolonged starvation may increase the rate of mitophagy and other types of organellophagy.

## Author contributions

S.T., I.K-H., and N.M. conceptualization; S.T. and C.S. data curation; S.T. formal analysis; I.K-H. and N.M. funding acquisition; S.T., C.S., I.K-H., and N.M. investigation; S.T., C.S., and I.K-H. methodology; I.K-H. and N.M. project administration; I.K-H. and N.M. supervision; S.T. visualization; S.T. writing-original draft; I.K-H. and N.M. writing-review and editing.

## Conflict of Interest

The authors declare that they have no conflicts of interest with the contents of this article.

## Abbreviations

3D: three-dimensional
ATG: autophagy-related
CLEM: correlative light and electron microscopy
DMEM: Dulbecco’s modified Eagle medium
EM: electron microscopy
ER: endoplasmic reticulum
FBS: fetal bovine serum
GFP: green fluorescent protein
HEK: human embryonic kidney
LAMP1: lysosomal-associated membrane protein 1
LC3: microtubule-associated protein 1 light chain 3 beta
MAM: mitochondria-associated ER membrane
MEF: mouse embryonic fibroblast
NRK: normal rat kidney
PAS: pre-autophagosomal structure
SBF: serial block face
SEM: scanning electron microscopy
